# Single blood-Hg samples can result in exposure misclassification: temporal monitoring within the Japanese community (United States)

**DOI:** 10.1186/1476-069X-11-37

**Published:** 2012-06-07

**Authors:** Ami Tsuchiya, Rob Duff, Alan H Stern, Jim W White, Finn Krogstad, Thomas M Burbacher, Elaine M Faustman, Koenraad Mariën

**Affiliations:** 1 Department of Environmental and Occupational Health Services, University of Washington, Seattle, WA, USA; 2 Washington State Department of Ecology, Olympia, WA, USA; 3 New Jersey Department of Environmental Protection, Trenton, NJ, USA; 4 Washington State Department of Health, Olympia, WA, USA; 5 Institute for Risk Analysis and Risk Communication, University of Washington, Seattle, WA, USA

**Keywords:** Uncertainty, Exposure, Fish, Blood, Mercury, Longitudinal, Methylmercury

## Abstract

**Background:**

The most prominent non-occupational source of exposure to methylmercury is the consumption of fish. In this study we examine a fish consuming population to determine the extent of temporal exposure and investigate the extent to which single time estimates of methylmercury exposure based on blood-Hg concentration can provide reliable estimates of longer-term average exposure.

**Methods:**

Blood-mercury levels were obtained from a portion of the Arsenic Mercury Intake Biometric Study (AMIBS) cohort. Specifically, 56 Japanese women residing in the Puget Sound area of Washington State, US were sampled on three occasions across a one-year period.

**Results:**

An average of 135 days separated samples, with mean blood-mercury levels for the visits being 5.1, 6.6 and 5.0 μg/l and geometric means being 2.7, 4.5 and 3.1 μg/l. The blood-mercury levels in this group exceed national averages with geometric means for two of the visits being between the 90^th^ and 95^th^ percentiles of nationally observed levels and the lowest geometric mean being between the 75^th^ and 90^th^ percentile. Group means were not significantly different across sampling periods suggesting that exposure of combined subjects remained relatively constant. Comparing intra-individual results over time did not reveal a strong correlation among visits (r = 0.19, 0.50, 0.63 between 1^st^ and 2^nd^, 2^nd^ and 3^rd^, and 1^st^ and 3^rd^ sample results, respectively). In comparing blood-mercury levels across two sampling interval combinations (1^st^ and 2^nd^, 2^nd^ and 3^rd^, and 1^st^ and 3^rd^ visits, respectively), 58% (n = 34), 53% (n = 31) and 29% (n = 17) of the individuals had at least a 100% difference in blood-Hg levels.

**Conclusions:**

Point estimates of blood-mercury, when compared with three sample averages, may not reflect temporal variability and individual exposures estimated on the basis of single blood samples should be treated with caution as indicators of long-term exposure. Reliance on single blood samples can make predicting ongoing methylmercury exposure highly speculative due to the large intra-individual variability.

## Background

By far, the most important non-occupational pathway for methylmercury (MeHg) exposure is fish consumption [1]. None of the forms of mercury have a role in normal human physiological function. Children who have been exposed *in utero* to MeHg can develop neuropsychological deficits [1]. To better understand the exposure-effect relationship for MeHg within individuals or populations, indicators of exposure, such as mercury (Hg) concentrations in hair, toenails, maternal-blood, umbilical cord tissue and cord-blood, have been used.

These biological indicators have also been used to measure temporal variability in exposure. Repeated sampling of individuals over time offers the hope of capturing intra-individual variability including variations in consumption behavior resulting from seasonal differences in species availability, and episodic consumption of fish with high MeHg concentrations. Blood-Hg data for the general US population from 1999–2004 suggest that levels may be decreasing with time though the reasons for this decrease are unclear [2]. In an indigenous Canadian population followed across five years, hair-Hg levels were shown to have seasonal variation with the highest mean hair-Hg concentrations approaching 17 mg/kg and lowest mean values below 5 mg/kg within a one year cycle [3]. There have also been studies in which hair-Hg levels were examined across gestation or across a one year period within fish consuming populations [4-7]. Associations between Hg in blood and hair have also been examined within the same population at ages 7 and 14 [8]. Although these data have provided some insight into temporal variation in Hg body burden levels, multiple time estimates of MeHg exposure within a population using blood-Hg levels has not been examined. Further, most MeHg exposure data based on biological indicators have been derived from biological samples collected at only a single time point. As part of the Arsenic Mercury Intake Biometric Study (AMIBS), we examined longitudinal data for blood-Hg levels within the population of Japanese women living in the Puget Sound area of Washington State (US) at three time points. MeHg exposure is of importance as this population consumes substantially more fish than the national average [9,10]. The main goals of this work were two-fold: to examine temporal blood-Hg levels within this group and to quantify intra-individual blood-Hg concentration variability across a period of approximately one year. This information will allow for a better understanding of potential misclassification of exposure resulting from intra-individual variability in temporal blood-Hg levels.

## Methods

### AMIBS

Of the 106 Japanese women participants in AMIBS, this manuscript describes the results from 56 Japanese women who provided blood samples during all three clinic visits. In addition to the Japanese participants, AMIBS includes 108 Korean women participants but only the Japanese participant data are discussed as only they provided blood samples across all three visits.

Detailed descriptions of AMIBS have been published [9,10]. The study included women of childbearing age (18 – 45 years of age), who identified themselves as Korean, Japanese or, of Japanese or Korean descent, and who had lived in the Puget Sound area of Washington State, US, for at least 6 months. To participate, women had to be willing to provide a hair sample from the nape of the neck (0.5 cm in diameter) and participate in a fish consumption survey (FCS). Enrollees also completed a self-administered food frequency questionnaire (FFQ) and could provide additional biological samples (urine, blood and/or toenails) at their discretion.

The Japanese participants were interviewed three times across 14 months. On the 1^st^, 2^nd^ and 3^rd^ visits, data were obtained for 106, 90, and 85 individuals, respectively. Participants returning for 2^nd^ and 3^rd^ visits were interviewed in the same manner as during the 1^st^ visit except the recall time period for the FFQ and FCS was different between 1^st^ and 2^nd^/3^rd^ visits. This has previously been described but in brief, for the 1^st^ visit, the surveys were open-ended and individuals could respond to questions about their fish consumption behavior spanning a year-long period while for the 2^nd^ and 3^rd^ visits, information obtained from the surveys covered the last two weeks prior to the date of the visit; thus they were two-week recall surveys [7]. Individual estimates of fish and Hg intake have been described previously and these data were used in this study for comparative purposes with blood-Hg levels [9,10]. Briefly, a detailed FCS with pictorial representations of approximately 70 fish species with multiple names in three languages along with fish portion models for cooked and raw fish were used to derive species-specific intake data for each individual which when combined provided for an estimated total daily fish ingestion rate. Species specific-Hg levels were obtained from the literature and from lab analyses conducted to determine Hg concentrations from fish species (20 species analyzed) for which there were no or limited available data [9]. Specifically, fish commonly consumed by the Japanese and Korean populations in the Puget Sound area were purchased at local Asian markets and analyzed for total Hg. Estimated species specific fish intake and Hg fish tissue concentrations allowed for an estimated Hg ingestion rate to be determined for each individual. Informed consent was obtained from all study participants, and the study design and materials were approved by the State of Washington Department of Social and Health Services Human Research Review Board.

### Blood sampling and hg analysis

Preparation and analysis were carried out following method EPA SW 846 7471B [11]. Blood samples were drawn at the Nadeshiko Clinic at the time of the interview. EDTA evacuated tubes were used to obtain 15 mls of blood from each individual. Samples were placed on ice, and transported to a certified laboratory (AMTEST Inc., Redmond, WA) where they were stored at −10 C until analyses were conducted to determine levels for total Hg. Whole blood samples, blanks and certified reference materials were analyzed using cold-vapor atomic-absorption spectrometry, with a detection limit of 0.2 μg/l. Batch sample analyses were conducted reflecting the clinic visit periods and samples were run in duplicate and matrix spikes were run at a rate of 10%. The lack of precision for the method (coefficient of variation from repeated measurement of reference materials) was 5.8%, 7.6% and 5.4% at blood-Hg levels of 1.1, 15.7 and 31.7 μg/l, respectively.

### Statistical analyses

A repeated-measures one-way ANOVA was used to compare blood-Hg levels among sample intervals. Mean individual blood-Hg levels based on pregnancy status were determined and then compared using Student’s *t*-test. Also, mean individual blood-Hg levels adjusted for change in plasma volume during pregnancy were compared with mean levels from non-pregnant participants using Student’s *t*-test [5,12]. In addition, minimum and maximum blood Hg concentrations were identified for each individual and Student’s *t*-test was used comparing first the minimum and then the maximum Hg concentrations grouped by pregnancy status. Intra-individual variability in blood-Hg was investigated by examining observed absolute change across clinic visits and by testing if variability increased as the maximum blood-Hg levels increased. Pearson product–moment correlation coefficients were determined to compare blood-Hg levels with estimated intake values of fish and with estimated intake of Hg. In addition, these correlations were obtained with and without adjusting for actual individual body weights and pregnancy status. Statistical analyses were performed using a significance level of 5% (p < 0.05) using Stata (Stata Corporation, College Station, Texas), Excel (Microsoft Corporation, Redmond, Washington) and SPSS (SPSS Inc., Chicago, Illinois).

## Results

The results of the blood-Hg concentrations for the participants who completed three clinic visits (n = 56) are provided in Table 1. No significant difference in blood-Hg concentrations between pregnant (n = 22) and non-pregnant (n = 34) participants was observed (p < 0.05) when comparing means. Because the stage of pregnancy may affect maternal blood-Hg levels, the minimum and maximum blood-Hg levels were identified for each individual and compared by pregnancy status. Again, no significant difference was observed for either the lowest or the highest concentrations. In another analysis examining the influence of pregnancy on blood-Hg levels, blood-Hg levels were adjusted for blood volume based on time of gestation with no significant difference observed (p < 0.05) between pregnant and non-pregnant participants. The average number of days between visits was 135 (± 19) days with the shortest interval being 100 days and the longest, 229 days. The interquartile range was 125 to 143 days. The mean time period spanned by the three visits was 270 days. The blood-Hg levels of these Japanese fish consumers were compared with the 2003–2006 National Health and Nutrition Examination Survey (NHANES) blood-Hg data which reflect the US as a whole (Table 1) [13]. Mean values for the Japanese participants greatly exceed the national average. Geometric mean and median blood-Hg values for the 2^nd^ and 3^rd^ visits of the Japanese participants were between the 90^th^ and 95^th^ percentiles of the levels observed in NHANES. The 1^st^ visit geometric mean and median values fell between the 75^th^ and 90^th^ percentile of the national distribution. The arithmetic mean values for each of the three visits were close to the 5.8 μg/l value associated with a MeHg uptake equivalent to the RfD [14].

**Table 1:** Blood-Hg level (μg/l) comparison between Japanese from three clinic visits with general US population data

				**Percentiles**					
	**n**	**GM**	**Mean**	**10th**	**25th**	**50th**	**75th**	**90th**	**95th**
1st Visit	56	2.66	5.10	1.28	1.40	1.84	3.77	10.85	26.14
2nd Visit	56	4.46	6.55	2.50	3.23	4.40	7.25	12.10	19.13
3rd Visit	56	3.10	5.00	1.25	2.66	3.78	5.23	9.15	14.25
NHANES 2003-06	8556	0.82	NA^a^	0.23	0.40	0.80	1.60	3.02	4.47

Mean, geometric mean (GM) and percentile values depicted.

^a^ NA, not available.

**Figure 1:**
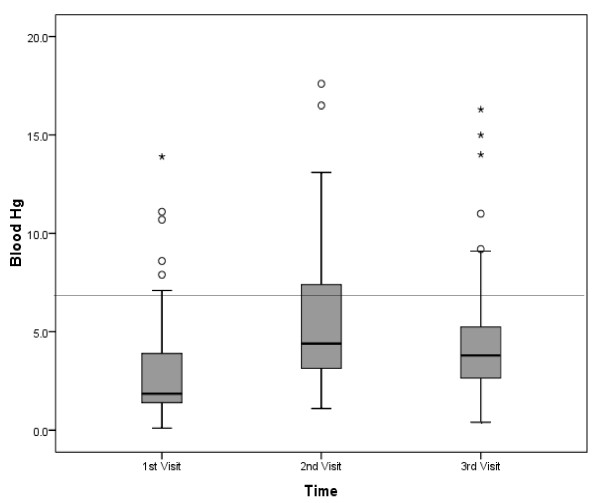
**Blood-Hg levels for each visit (n = 56).**^**a**^ Blood-Hg values in μg/l. Medians are middle lines within box. Top and bottom of box represents upper and lower quartile values, respectively. Upper and lower whiskers represent sample maximum and minimum values, respectively. Values marked with “°” and “*” are outliers as defined by being 1.5x and 3x the interquartile range, respectively. Four (25.1, 29.3, 35.0 and 44.3), three (23.7, 24.3 and 28.2) and one (33.9) blood-Hg values are not depicted for the 1^st^, 2^nd^ and 3^rd^ visits, respectively, as they are off the scale provided. Distribution mean values do not differ significantly (p <0.05). Horizontal line represents 5.8 μg/l blood-Hg (the RfD equivalent).

**Figure 2:**
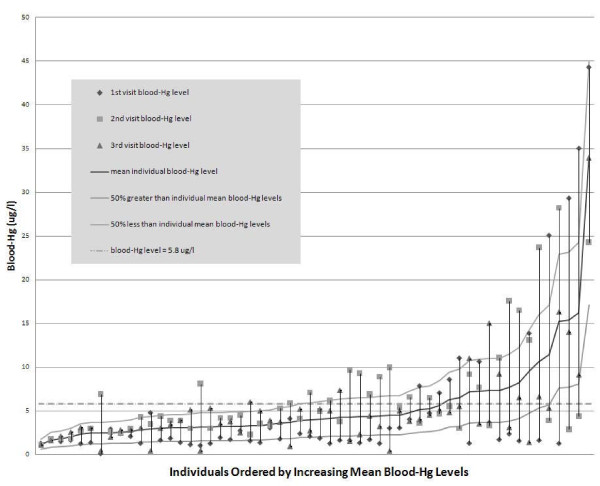
Intra-individual blood-Hg variability across three successive clinic visits depicting exposure and pharmacokinetic variability.

Distributional data of blood-Hg levels for all clinic visits are presented as box-plots in Figure 1. There was no significant difference (p <0.05) in blood-Hg levels observed among the three sampling visits. However, the relatively small sample limited the power to detect differences in the blood-Hg levels at the three sampling visits. The means for each sample period (5.1, 6.6 and 5.0 μg/l) were not significantly different. Respective 95^th^ percentile confidence intervals were 2.25, 1.49 and 1.35, and coefficients of variation were 1.7, 0.87 and 1.0. Though means of the distributions of these participants did not change significantly across sampling periods, many individual blood-Hg levels varied by 100% or more. The absolute change in blood-Hg levels on an individual basis between successive samples across the three visits is depicted in Figure 2.

Evidence of a strong monotonic agreement among visits was not discernable as the number of individuals with increasing blood-Hg levels between the 1^st^ and 3^rd^ visit was 35 (63%) and between 1st and 2nd and 2nd and 3rd was 42 (75%) and 24 (43%), respectively. Correlations between all three groups were weak to moderate (r = 0.19, 0.50, 0.63 between 1^st^ and 2^nd^, 2^nd^ and 3^rd^, and 1^st^ and 3^rd^ sample results, respectively; p < 0.05 for all). These modest correlations and the lack of a strong agreement were reflected in the variability observed in blood-Hg levels in individuals across the three successive clinic visits during the study period (Figure 2). For 58% (n = 34), 53% (n = 31) and 29% (n = 17) of the individuals there was a minimum 100% change in blood-Hg levels between visits (1^st^ and 2^nd^, 2^nd^ and 3^rd^, and 1^st^ and 3^rd^ visits, respectively). In total, there were 82 blood-Hg measurements taken at two different time points that were separated by at least a two-fold difference and of these, 27 (33%) decreased between sampling time points while 55 (67%) increased. Of the 10, 21 and 11 individuals that exceeded 5.8 μg/l of blood-Hg on the 1^st^, 2^nd^ and 3^rd^ visits, respectively, only one individual remained consistently above this RfD equivalent for exposure during the study period. All other individuals that exceeded 5.8 μg/l had at least one sample value that was below the 5.8 μg/l level. Percent differences from the individual mean values in blood-Hg concentrations for each individual, as determined by dividing each sample concentration by the three-sample average (from the three visits), ranged from −95% to +179%. The single sample estimates had an average observed difference of 44% when compared with the three-sample average and only 61% of the samples (103 of 168) were within 50% of the three-sample average.

Intra-individual variability was examined as a function of blood-Hg concentration to test if variability increased as the maximum blood-Hg levels increased. Individual maximum blood-Hg concentration was plotted against the difference between the individual’s maximum and minimum blood-Hg concentrations as a percentage of the maximum blood-Hg concentration. Variability was linear across the blood-Hg concentrations observed in this study (r = 0.42) although sensitivity analyses indicated that those individuals with the highest maximum (and mean) blood-Hg levels (mean >10 μg/l; n = 6; Figure 2) had greater intra-individual variability. Variability decreased when omitting these individuals resulting with markedly improved correlation of r = 0.64 (data not shown).

**Figure 3:**
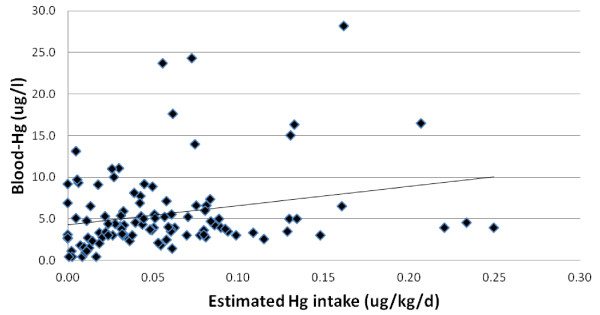
**Example of correlation between estimated Hg intake based on food consumption survey and blood-Hg levels.** Example is of data from the 2^nd^ and 3^rd^ visits (r = 0.25). For all correlation analyses conducted between Hg intake and blood levels across the study period, no correlation coefficient exceeded 0.3 and regression models did not fit the data (all r^2^ < 0.1).

Blood-Hg levels were compared with estimated intake values of fish and Hg with and without controlling for individual body weight as well as pregnancy status across the clinic visits. An example of correlation is provided in Figure 3 which depicts data from the 2^nd^ and 3^rd^ visits combined for all participants such that each individual provides two data points; for each visit, individual blood-Hg values are plotted against FCS derived data that estimated individual Hg intake on a per kilogram body weight basis. Correlation was based on individual mean values of the two measurements and was not strong (r = 0.25, r^2^ = 0.06, p < 0.05). For all analyses conducted across the given time periods, correlation coefficients did not exceed 0.3 and no r^2^ value exceeded 0.1 (p < 0.05).

## Discussion

To better define dose–response associations linking MeHg exposure with deleterious effects, whether the effect be neurodevelopmental or cardiovascular, it is important to reduce uncertainty and address the limitations of exposure assessments. The relationship between dose and response may be obscured when biomarker values are unable to adequately reflect ongoing exposure. Single time point measurements are subject to a range of factors (e.g. variations in consumption frequency, magnitude and duration) that can lead to uncertainty when using single samples to estimate average MeHg exposure over time [15]. In addition, since biological samples integrate MeHg exposure over variable periods of time, the use of such measures can result in uncertainty when attempting to estimate exposure over a specific period of time (e.g., a single trimester of gestation) [1]. These considerations become particularly important when MeHg biomarkers are employed to reconstruct the intake dose associated with an observed biomarker-outcome relationship such as those observed in the Faroe Island study. Such reconstructions are required in order to convert such data into fish consumption advisories [16]. This work suggests that, on an individual basis, blood-Hg levels from separate exposure time periods do not provide a reliable estimate of longer term exposure as represented by a three sample average.

Single time point blood-Hg analysis reflects the aggregate of recent as well as less recent exposure periods. Blood samples were obtained so that the measured Hg levels represented exposure periods largely independent of each other with respect to the half-life of MeHg in blood. The half-life of MeHg in blood is considered to be approximately 50 days and more than two half-lives separated each participant’s samples in this study [17,18]. Accordingly, less than 25% of an individual’s prior mercury exposure will be reflected in the blood-Hg levels observed between successive samples. This tended to minimize (but not eliminate) auto-correlation among the samples. The similarity in the pooled distribution of blood-Hg values between sampling events, based on group mean values that did not differ over time, is consistent with previous observation made by Tsuchiya et al. [7] in hair samples. As the individual sample values were not constant over time, the sum of intra-individual variabilities across the sampling periods appear to regress to the sample mean.

In contrast to the temporal stability of the three blood-Hg levels for the group as a whole, there was marked change over time in individual blood-Hg levels. Blood data show that separate and nearly independent samples obtained within a time period of several months can fluctuate by more than 100% and individual sample values exceed the individual’s average across the specified time period by greater than 50%. Further, the proportional variability in blood-Hg was similar for individuals at all levels of blood-Hg with the exception of those above the 90^th^ percentile in blood-Hg. These data may reflect both intra-individual pharmacokinetic variability as well as the change in MeHg intake observed across sample intervals. Accordingly, the temporal blood-Hg variability may reflect short-term changes in MeHg intake, absorption, distribution, metabolism, and elimination.

The temporal changes in blood-Hg levels may aid in explaining the large observed disparity in hair to blood ratios (mg Hg/kg hair: μg Hg/ml blood) found in the literature that range from less than 100:1 to approximately 400:1 [1]. A ratio of 250:1 is generally accepted for individuals having reasonably constant exposure levels. Yet, within this group of Japanese women, the ratios of the proximal 6 cm hair strand mean Hg levels (described previously, [7]) to the whole blood mean Hg levels across the three visits were 373:1, 242:1 and 300:1. The overall three-sample mean was 305:1.

There are two shortcomings with this work that need be noted. First, comparisons between mean blood-Hg levels, mean blood-Hg levels adjusted for change in blood volume during gestation, as well as minimum and maximum levels led to no observable difference by pregnancy status. Although no data exist from other sources that permit a comparison of these two groups, pregnant women have a decrease in Hg body burden between the 2^nd^ and 3^rd^ trimester possibly due to increased blood volume or transfer of Hg across the placental barrier to the fetus [5,12]. Our study was not developed to address this issue as participants were accepted into the study at various time periods during gestation with several women becoming pregnant during the study period resulting in only a few women being enrolled for the last two trimesters of pregnancy. Nonetheless, it seems reasonable that any pregnancy-related difference in blood Hg concentration was obscured by the overall intra-individual temporal variability. Further, the small sample does not provide for sufficient power to observe a difference between groups or to see a significant decrease in body burden across the last two trimesters of pregnancy. Second, since samples were analyzed for total blood-Hg, the results do not delineate between organic and inorganic forms of Hg. We cannot exclude exposure through inhalation (Hg vapor from dental amalgams), ingestion (drinking-water and food), or even from medical treatments, as well as possible contribution from indoor air [1,19,20]. Also, there is the ritual use of Hg in folk medicine within several populations that may allow for exposure to elemental or inorganic Hg salts [20].

These additional exposures could have impacted blood-Hg levels and affected correlations between fish consumption and blood-Hg levels. Comparisons between blood-Hg and the FCS reflecting dietary practices across the preceding 14 days provided for low correlations. Although the FCS was a robust instrument with pictorials and species names in multiple languages and used fish models for determining portion size, the estimated intake data did not provide for a precise representation of actual Hg intake [9. 10]. Not all fish species consumed by this population were analyzed for Hg. For the species not analyzed directly, literature based estimates of species specific Hg levels were used. These values may not have accurately reflected the same species or may not have reflected regional differences among the same species. Other factors that could have impacted the relationship between estimated Hg intake and body burden levels include recall bias, variability in toxicokinetics, nutritional status and dietary interactions [1,21-23].

The results of this work brings into question the use of blood-Hg samples collected at a single point in time to predict average exposure and risk. Physicians have been advised to obtain blood-Hg levels in individuals considered to be exposed to MeHg at elevated levels due to their fish consumption behavior patterns [24]. Although blood analysis may be one tool for examining ongoing exposure, assuming that symptomatic individuals will show elevated blood-Hg concentrations based on a single sample may lead to misdiagnosis and misclassification of exposure. Even within groups or populations that appear to have Hg body burden levels in steady-state, exposure periods largely independent of each other suggest that individual blood-Hg levels can vary markedly. In addition, as a single blood sample may be a relatively poor predictor of future exposure, a blood sample obtained for example at or before conception may not provide the best foundation from which to offer pre-pregnancy advice on ways to reduce exposure during the neurodevelopmentally sensitive stages in fetal development or to provide exposure estimates for use in epidemiologic studies.

If further research endeavors using, for example, a more robust sample size provide results supporting those observed in this study, then average long term Hg exposure values obtained from hair strands may provide an alternative to blood-Hg levels. Hair-Hg values may provide individuals overexposed or concerned about elevated exposures along with pregnant women or women who are considering pregnancy a better means from which to derive subsequent diet alterations so as to minimize MeHg exposure. There are concerns with the use of hair as a metric of exposure such as ethnic differences and impact from various available hair treatments as well as airborne-Hg deposition, but this type of strategy could allow entire populations with potentially elevated levels of exposure to MeHg to be protected through screening as analyses of hair for Hg using atomic absorption spectrometers have come with decreased costs and increased efficiency during the last decade.

## Conclusion

The blood-Hg data obtained for this group of Japanese women living in Washington State support the finding that these individuals are among the most exposed in the US and reflect results from hair-Hg data indicating that MeHg body burdens for this group may be stable or changing slowly across the study period. However, individual exposures estimated on the basis of single blood samples should be treated with caution as indicators of long-term exposure, as reliance on such spot blood samples make the prediction of ongoing MeHg exposure highly uncertain due to the large intra-individual variability.

## Abbreviations

AMIBS, Arsenic Mercury Intake Biometric Study; EPA, Environmental Protection Agency; FCS, Fish consumption survey; FFQ, Food frequency questionnaire; GM, Geometric mean; Hg, Mercury; MeHg, Methylmercury; NHANES, National Health and Nutrition Examination Survey; RfD, Reference Dose; US, United States; WA, Washington State.

## Competing interests

All authors declare they have no actual or potential competing financial or non-financial interests in this work.

## Authors’ contributions

AT was a significant contributor to many facets of AMIBS and was responsible for many others. AMIBS would not have been conducted in the exemplary manner that it was without her. RD made substantial contributions to the acquisition of data for AMIBS and was involved in drafting the manuscript. AS aided in drafting the manuscript and, in data analyses and interpretation of data. JWW provided substantial contribution to the development of the manuscript and in data interpretation. FK provided significant effort to data analyses and the interpretation of study results. TMB contributed significantly to study design and data interpretation. EM aided in the writing of the manuscript and contributed to the acquisition of the data. KM performed statistical analyses and aided in drafting the manuscript. All authors contributed substantially to the discussion of the data and their analyses, provided editorial comments to the draft manuscript and all approved the final manuscript.
